# Integrative analysis of pathway deregulation in obesity

**DOI:** 10.1038/s41540-017-0018-z

**Published:** 2017-06-30

**Authors:** Francesc Font-Clos, Stefano Zapperi, Caterina A.M. La Porta

**Affiliations:** 1 ISI Foundation, Via Chisola 5, 10126 Torino, Italy; 2 Center for Complexity and Biosystems, Department of Physics, Via Celoria 16, 20133 Milano, Italy; 3 CNR-Consiglio Nazionale delle Ricerche, Istituto di Chimica della Materia Condensata e di Tecnologie per l’Energia, Via R. Cozzi 53, 20125 Milano, Italy; 4 Department of Applied Physics, Aalto University, P.O. Box 11100, FIN-00076 Aalto, Finland; 5 Center for Complexity and Biosystems, Department of Environmental Science and Policy, University of Milano, via Celoria 26, 20133 Milano, Italy

## Abstract

Obesity is a pandemic disease, linked to the onset of type 2 diabetes and cancer. Transcriptomic data provides a picture of the alterations in regulatory and metabolic activities associated with obesity, but its interpretation is typically blurred by noise. Here, we solve this problem by collecting publicly available transcriptomic data from adipocytes and removing batch effects using singular value decomposition. In this way we obtain a gene expression signature of 38 genes associated to obesity and identify the main pathways involved. We then show that similar deregulation patterns can be detected in peripheral markers, in type 2 diabetes and in breast cancer. The integration of different data sets combined with the study of pathway deregulation allows us to obtain a more complete picture of gene-expression patterns associated with obesity, breast cancer, and diabetes.

## Obesity transcriptomic signature shares features with cancer

The worldwide increase in obesity is extremely worrisome, especially because this condition is associated with a higher risk for diseases such as type 2 diabetes and cancer. Identifying alterations in regulatory and metabolic activities associated with obesity is complicated due to the presence of noise. A team lead by Caterina La Porta from the University of Milan addressed the question from the point of view of big data and extracted a signature of 38 genes associated to obesity from the combination of publicly available gene expression data from obese and lean subjects. The results revealed a similarity between the deregulation patterns observed in obesity and those found in breast cancer and diabetes, providing a clearer picture of the role of obesity in these diseases.

## Introduction

Obesity is increasing worldwide, with impressive data showing that about 10% of children are overweight or obese in USA and Europe. From the medical point of view, obesity is overtaking smoking as the leading cause of premature death. The risk of many diseases, including cancer, autoimmune diseases and type 2 diabetes, is increased in obese subjects.^1^ In fact, obesity contributes in about more than 70% of diabetes cases^2^ and it has been seen associated to some types of tumors, such as breast cancer.^3^

Well established cases of Mendelian forms of obesity approximately account for only 5% of the severely obese cases.^4^ In the case of common obesity, recent genome wide association studies have investigated possible relations between single nucleotide polymorphism and Body Mass Index (BMI).^5^ Despite the sheer amount of data and the effort devoted to the task, none of the resulting genetic loci have real predictive power. In particular, genetic contributions do not account for most BMI variations between subjects which are likely due to lifestyle and environmental factors.^5^

A more refined and complete picture of the genetic aspects associated with obesity can be obtained by integrative approaches.^6–9^ For instance in ref. 6, the authors employed a method combining gene-expression and DNA variations to discover drivers of complex traits. In this way, the authors were able to identify and validate in mice new genes involved in susceptibility to obesity.^6^ Other results are based on network analysis^8^ and allowed identification of genes and metabolic pathways associated with obesity in mice.^9^

While there is clinical and epidemiological evidence of a link between obesity and some types of cancer, there is still not a robust gene expression signature pointing in this direction. Gene expression data provides a vivid picture of the alteration in regulatory activities taking place in cells, and finding a transcriptomic signature would help to better understand the relationship between obesity and cancer. To this end, several distinct studies have reported transcriptomic data in cells derived from a limited set of subjects with reported BMI, highlighting genes with significant differences in expression level.^10–18^ However, due to the high variability between patients and the limits of in vitro models, a clear picture of a possible signature is still not available. An important factor for success in this task is to reduce the massive amount of noise which is unavoidable in any transcriptomic data set: Typical studies have access to a limited numbers of samples, in the 10–10^2^ range, and try to reveal a clear signature from a large set of genes, typically in the order of 10^4^. Finding significant patterns in a large dimensional and noisy data set is complicated and often leads to large differences in the results reported in each study.

Here, we propose to alleviate the noise problem in gene expression data by combining different data sets obtained from the literature. Extracting useful information by merging data sets stemming from different experiments is, however, a challenging task due to *batch effects*: each experiment introduces a bias in the data that is due to technical processing and unrelated to biological factors. This systematic source of variation masks any biological differences when comparing samples coming from distinct batches. In the present paper we eliminate batch effects using the method of singular value decomposition (SVD)^19^ and further we reduce the noise by computing pathway deregulation scores (PDS) for the resulting data.^20^ The combination of these two steps allows for a dramatic noise reduction and reveals gene expression patterns that would otherwise be inaccessible when focusing on individual genes in a single batch. Using this approach, we find a robust signature of 38 genes with a statistical significance of 5*σ*, the confidence level required in particle physics to announce a discovery, that is able to discriminate between obese and lean subjects from adipocyte transcriptomes. We can associate this signature to a single score that correlates very well with BMI also in other independent transcriptomic data.

From the biological point of view, the 38 genes of the signature are interesting: it includes genes involved in the interaction between cells and the extracellular matrix and factors involved in inflammation. The signature includes also genes involved in typical correlated adverse symptoms of obese subjects, such as those linked to the central nervous system, the digestive system and fertility.

Next, we investigate our transcriptomic signature by comparing data from breast cancer tissue with healthy breast tissue and find similar pathway deregulation in breast cancer and obesity, confirming the strong association between the two. Our score also correlates very well with diabetes in subjects with similar BMI. Furthermore, we investigate if we are able to find the same signature using the transcriptomes obtained from monocytes.

Finally, we have also investigated if bariatric surgery is able to affect gene expression profiles associated with the signature both in adipocytes and monocytes. Our analysis clearly shows that bariatric surgery does not affect gene expression, at least after 3 months. In sum, our signature provides a complete picture of gene expression in obesity, breast cancer, and diabetes, suggesting possible interesting targets for therapeutic intervention and indicating that adipocytes are a reliable tissue for clinical studies in obese subjects.

## Results

### Transcriptomic signature of obesity in adipocytes

**Fig. 1:**
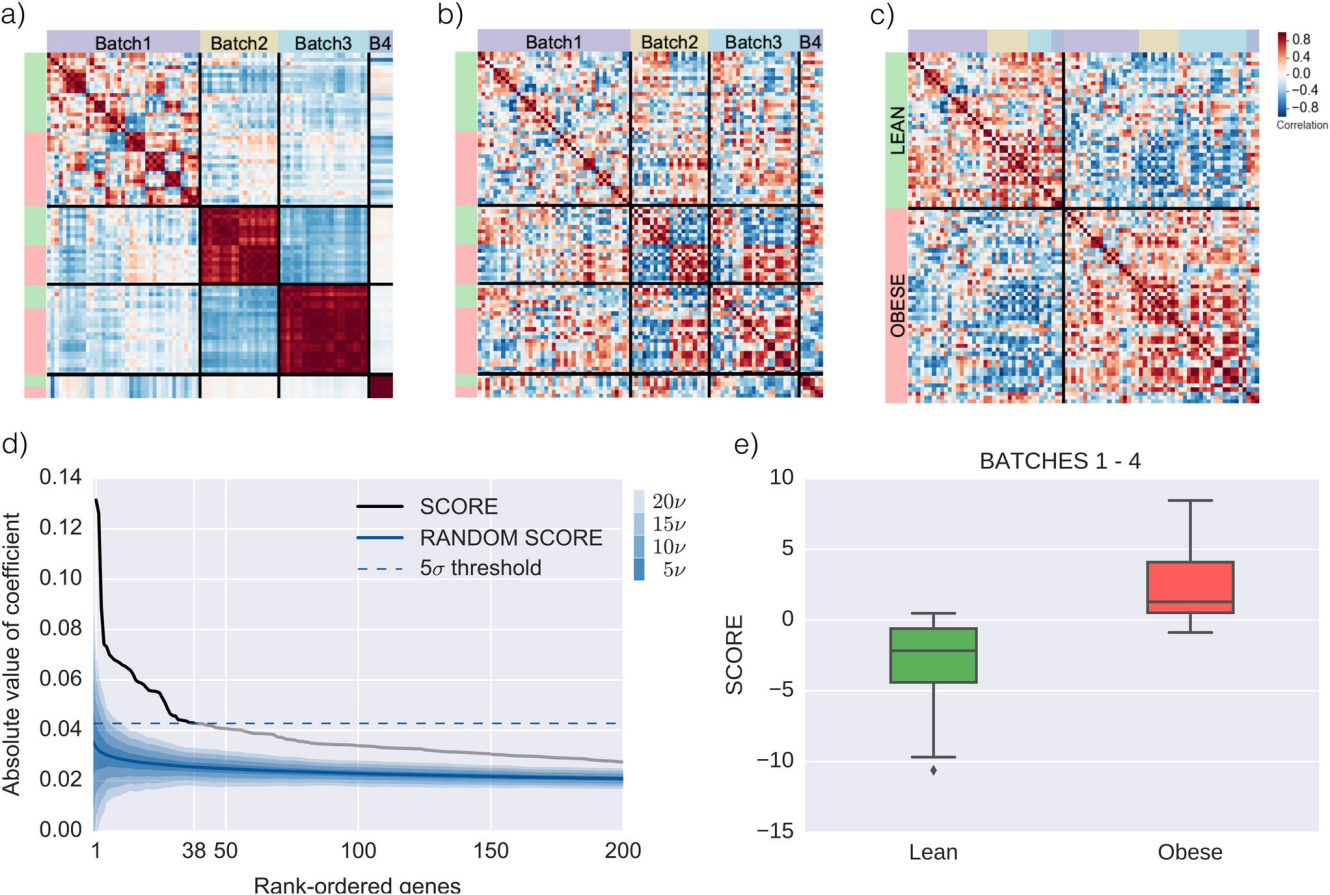
Merging different data sets leads to a strong signature. **a**, **b**, **c**: Visualization of the batch-effects removal process. Heatmaps showing correlations among samples before **a** and after **b**, **c** the application of our two-step singular value decomposition (SVD) filtering process, see Methods for details. *Red* indicates positive correlation, while *blue* indicates negative correlations. Samples are grouped by batch in panels **a**, **b** and by Body Mass Index (BMI) status in panel **c**. Correlations are computed using only the first seven principal components to enhance visualization. **d** Significance of the obesity score coefficients. The 200 genes with highest coefficient (in absolute value) in the first principal component of Batch1–4 after SVD filtering (*black* and *gray line*) and in a random vector (*solid blue line*), see Methods for details. The *dashed blue line* marks the 5-*σ* significance threshold used to extract the 38 genes of our obesity signature. The 4 *blue shaded regions* (ranging from *darker*, more probable, to *lighter*, less probable), mark increasing intervals of 5*ν*, where *ν* is the standard deviation of each individual ranked gene in a randomized score. **e** Obesity score at different BMI status. Obesity score boxplots for patients categorized either as lean or obese for Batch1–4. Notice that this batch is also used to construct the score. For validation data, see Figure 2

We find a robust transcriptomic signature of obesity by integrating four adipose tissue gene expression data sets (batches 1–4, see Supplementary Table 4) via a two-step SVD filtering process. The technical details of this process are discussed in the Methods Section, but we briefly sketch its functioning here. Through the use of linear algebra operations (i.e., SVD filtering), it is possible to identify and remove most batch effects: the part of the variability of the data that is not of biological origin.^21^ Indeed, Fig. 1a shows that samples from the same batch are initially similar to each other (marked with *dark red coloring* corresponding to high correlations), while samples from different batches are different from each other (*white* or *blue coloring*, corresponding to no correlation or anti-correlation, respectively). Due to this batch effect it would not be possible to simply merge batches 1–4. After applying our SVD-filtering method, however, batch-effects are mostly eliminated and samples do not cluster by batch any more (see Fig. 1b), but instead by BMI value (Fig. 1c): samples with the same BMI value tend to be correlated (*red coloring*), while samples with different BMI values tend to be anti-correlated (*blue coloring*).

These correlations, however, are a combination of the expression of all genes. To further investigate which genes are most responsible for these correlations, we compute the first principal component of the merged data, which in practice is a vector where each gene has a coefficient. We then rank genes by the absolute value of their coefficient, compare with the same procedure applied to a random vector, and select those genes whose coefficient is above a 5*σ* threshold (FDR equivalent: 1.90 × 10^−3^), as shown in Fig. 1d. In this way, we identify 38 genes and their associated coefficients as a transcriptomic signature of obesity. We compare our coefficients with the BMI-association summary statistics released from the Twins UK dataset in ref. 22, and verify that all genes except one in the obesity score show changes in the same direction (up/down regulated) in the Twins UK dataset, see Supplementary Figure 2. In summary, to each adipose tissue sample we can assign an obesity score, defined as a linear combination of the (log_2_) expression of the genes in the signature.

Supplementary Figure 1 summarizes our results, ranking all the 38 genes in the signature in terms of fold change of their expression with respect to the control case (i.e., obese vs. lean). We also compare our results with the original papers where individual batches where studied. We can see that while some of the genes were discussed in some of the papers, the most significant genes we found here were not the focus of those papers and were mostly just mentioned in the supplement. The largest overlap is found with the results of ref. 10 where, however, over 600 genes were reported as significantly changed.

**Fig. 2:**
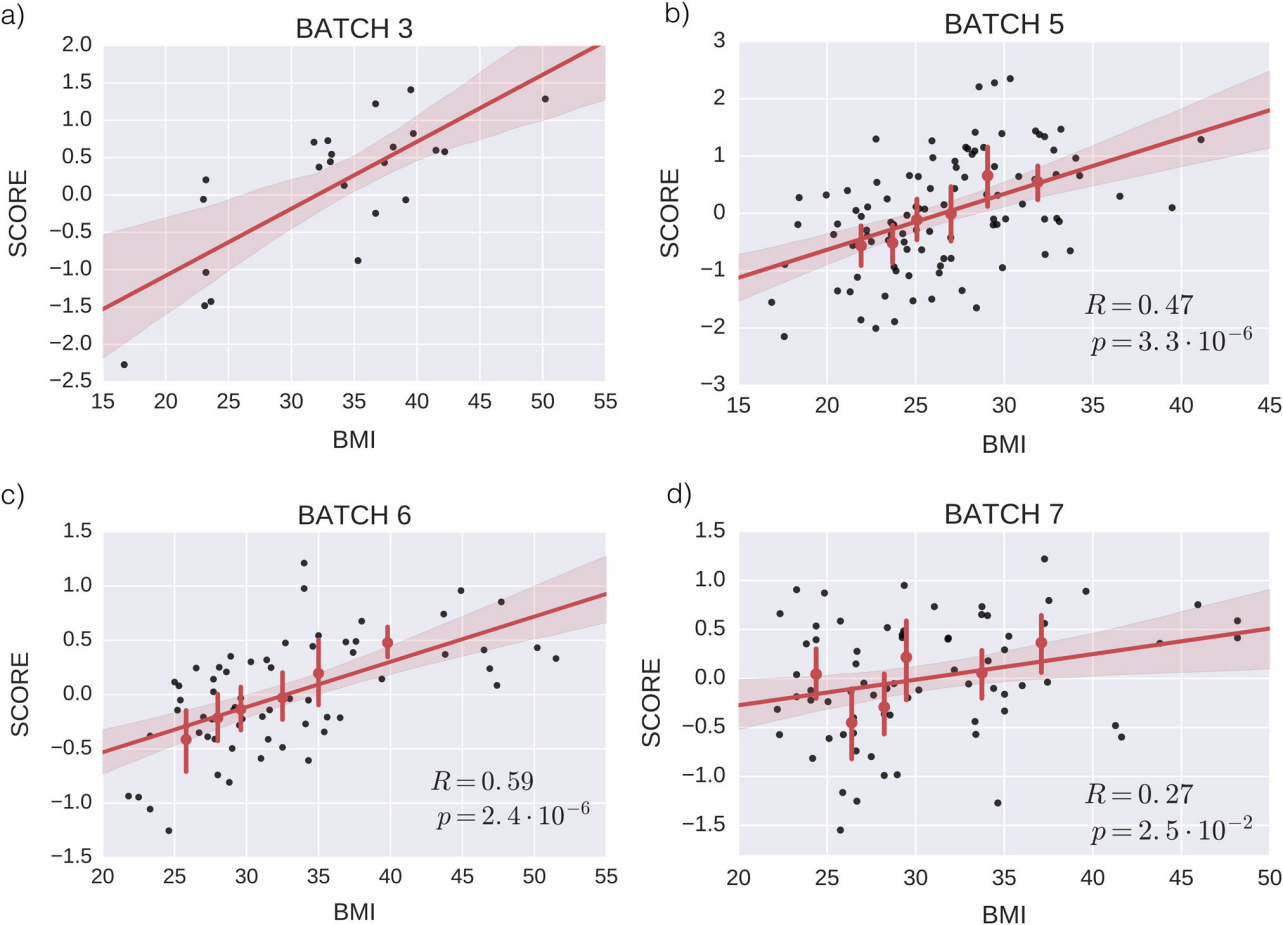
The obesity score correlates with BMI. Scatter plots (*black solid dots*) and linear least-square regression fits (*red lines*) of the obesity score against BMI, for batch 3 **a**, batch 5 **b**, batch 6 **c** and batch 7 **d**. *P*-values are computed for validation data sets via two-sided null hypothesis of 0 slope. Batch 3 was also used to construct the score and is shown only as reference, while batches 5, 6, and 7 are independent validation data sets. Data is binned using percentiles and displayed with 95% confidence intervals (CI) *red error bars*, with a *red dot* marking the mean value of each bin. The *red solid line* is the fitted regression line, and the *red shaded area* corresponds to 95% CI of the regression line, computed via bootstrap.

A key feature of our approach is that the coefficients of the obesity score (Table 1) are not the result of a fit that yields the best correlation with BMI in the training data (batches 1–4). Instead, the lean/obese categorical information is used to choose which eigengenes to filter out (see Methods for details), but the values of the coefficients are the result of plain SVD. This is a technical but crucial point, because it implies that our methodology is less prone to suffer from over-fitting issues, and hence renders the signature highly robust. To test this claim, we gather additional validation data sets (batches 5–7), totaling *N* = 238 validation samples, and compute its obesity scores. Figure 2 displays our main result: Remarkably, the obesity score is well-correlated with BMI in batch 6 (*R* = 0.59, *p* = 2.41 × 10^−6^) and batch 5 (*R* = 0.47, *p* = 3.29 × 10^−6^), and moderately correlated with BMI in batch 7 (*R* = 0.27, *p* = 2.48 × 10^−2^). The correlation with batch 3 is shown only for comparison, as batch 3 is part of the data used to construct the score and hence cannot be used to validate the score.

**Table 1:** The 38 genes in the obesity score, and their associated coefficients

Rank	Entrez ID	Gene symbol	Coefficient	Rank	Entrez ID	Gene symbol	Coefficient
1	1278	COL1A2	0.131414	20	7045	TGFBI	0.056923
2	80763	SPX	−0.126199	21	25878	MXRA5	0.055820
3	761	CA3	−0.088910	22	2982	GUCY1A3	0.055620
4	219348	PLAC9	0.074152	23	2335	FN1	0.055548
5	25975	EGFL6	0.073139	24	7076	TIMP1	0.055335
6	2014	EMP3	0.070109	25	5396	PRRX1	0.054843
7	6696	SPP1	0.068951	26	4069	LYZ	0.052908
8	1397	CRIP2	0.067884	27	8076	MFAP5	0.051032
9	1490	CTGF	0.067408	28	3512	JCHAIN	0.048567
10	22822	PHLDA1	0.066730	29	10402	ST3GAL6	−0.046569
11	1880	GPR183	0.065863	30	3429	IFI27	0.045810
12	171024	SYNPO2	0.065466	31	83442	SH3BGRL3	0.045708
13	1520	CTSS	0.064611	32	712	C1QA	0.044201
14	80114	BICC1	0.063828	33	474344	GIMAP6	0.044113
15	115207	KCTD12	0.062233	34	9457	FHL5	0.043849
16	151887	CCDC80	0.059890	35	8470	SORBS2	0.043746
17	22918	CD93	0.059141	36	7037	TFRC	0.043140
18	389136	VGLL3	0.058799	37	1291	COL6A1	0.042982
19	8542	APOL1	0.058107	38	57863	CADM3	0.042899

*Note*: Genes are ranked by the absolute value of the coefficient

**Fig. 3:**
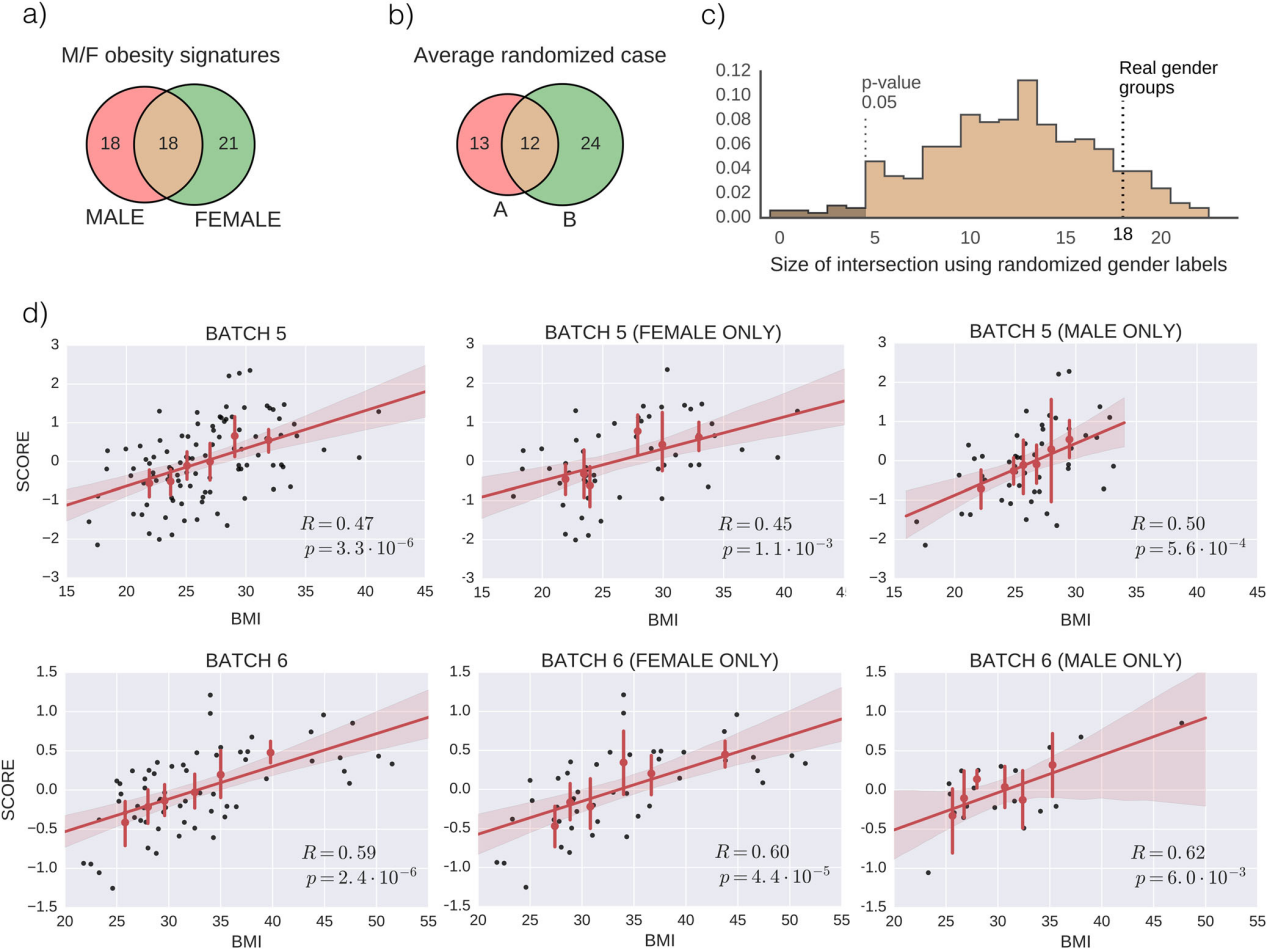
The obesity signature is not gender-dependent **a** Venn diagram of male/female signatures. **b** Venn diagram of a representative example of random A/B signatures, showing that the overlap obtained with male/female groups is qualitatively similar to that obtained with random A/B groups of the same sizes (see Methods Section in main text for details). **c** Null distribution of number of overlapped genes, showing that the overlap of 18 genes in the real male/female signature is compatible (*p* = 0.874) with the null hypothesis of gender-independent signature. **d** Scatter plots for batches 5 and 6, using all samples (*left column*), female-patient samples only (*middle column*) or male-patient samples only (*right column*). The figure shows that the association between the obesity score and BMI is statistically significant in both male-only and female-only populations (notice that the reported *p*-values in the male-only and female-only panels are lower than their all-gender counterparts, but this is due to the decreased number of samples).

Some of the batches we use in our analysis report also the gender of each subject (see Supplementary Table 4). We use those data to check if the signature we find is gender-specific by computing male-only and female-only signatures (see Fig. 3). Using the data of batches 1–4, we cannot reject (*p*-value 0.87) the null hypothesis of a gender-independent obesity score.

In summary, we extract a signature of obesity by merging batches 1–4 via a two-step SVD-filtering method, and validate it using batches 5–7. Our signature of obesity is composed of only 38 genes, and assigns a numerical obesity score to each sample. The obesity score gives high correlation with BMI in batch 3, and good correlations with BMI in batches 5–7.

### Pathway deregulation in obesity adipocytes and breast cancer tissues

**Fig. 4:**
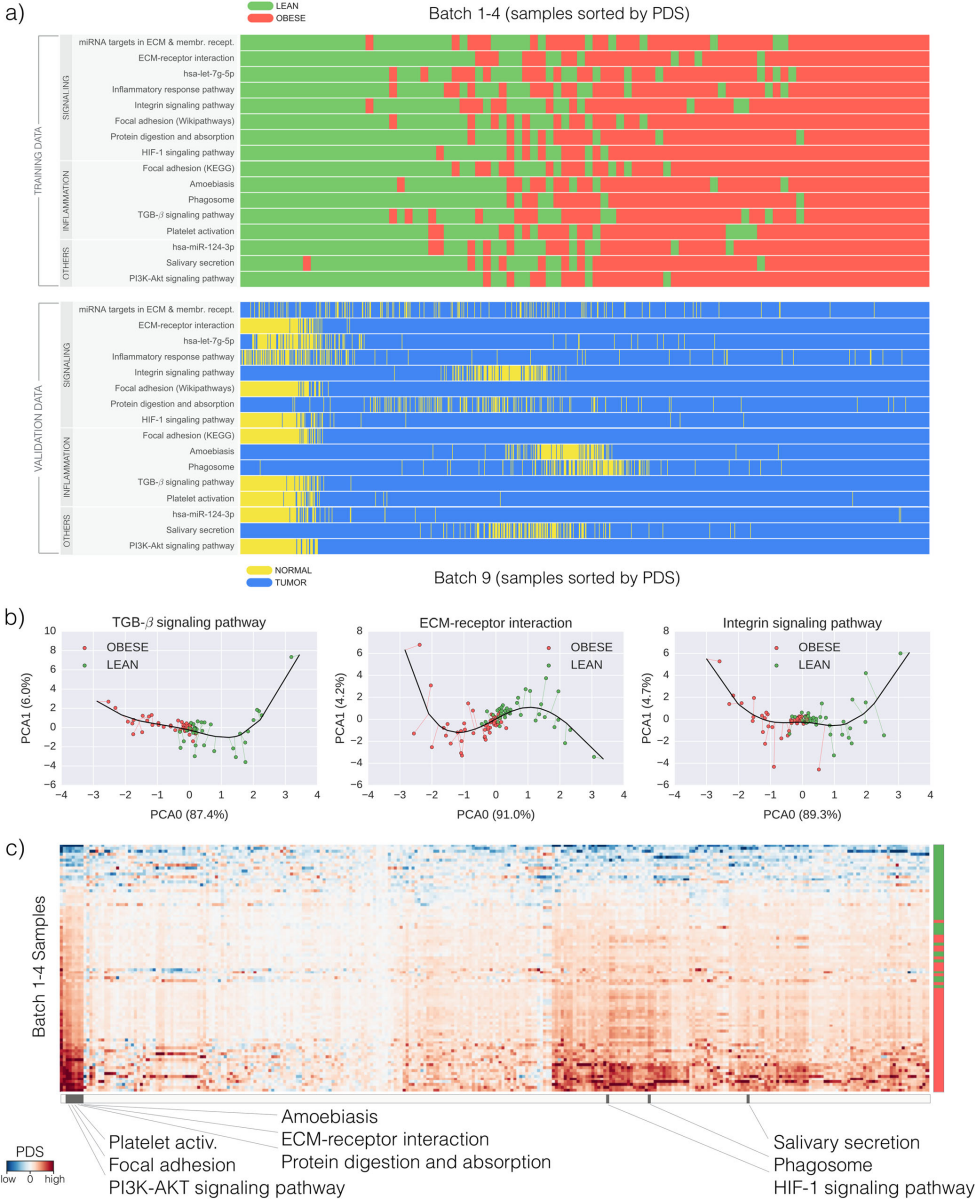
Deregulation of pathways in obese and cancer patients. **a** Samples sorted by Pathway Deregulation scores (PDS) display strong clustering both for obesity data (batches 1–4), as well as cancer data (batch 9). **b** Projection of batches 1–4 samples, shown with *red* (obese) and *green* (lean) *dots*, onto the principal curve (*black line*) that is used to define PDS, see Methods. We show three pathways (nine in Supplementary Figure 4) from the 16 found to be significantly over-represented in the 38 genes of the obesity score. For the purposes of this visualization only, all data is further projected onto its first two principal components, PCA0 and PCA1. Axis labels display the ratio of explained variance. **c** Heatmap for PDS of all KEGG pathways. Each pixel represents the value of the PDS (from *blue*, low PDS, to *red*, high PDS) for a each sample (row) and pathway (column). Pathways are hierarchically clustered according to similarity of expression in obesity samples. Samples are sorted by their obesity score. *Green*/*red labels* in the *right* indicate lean/obese categories. *Black labels* in the *bottom* mark the nine KEGG pathways from the 16 selected using the obesity score. The panel shows that these pathways tend to cluster together and are among the most highly deregulated.

We perform gene set over-representation analysis to identify relevant pathways using the 38 genes of the obesity signature and pathways from several databases, see Methods for details. In this way, we obtain a list of 16 pathways that contain at least two genes from the signature (unadjusted *p*-values range from 1.90 × 10^−7^ to 3.47 × 10^−3^, see Supplementary Table 3. Family-level *p*-value for the set of 16 pathways equals 0.012, see Methods for details). We then compute PDS for these 16 pathways (nine shown in Fig. 4) using both batches 1–4 as well as batch 9, which corresponds to the breast cancer cohort of TCGA.^23^ In short, PDS are a way to quantify the global deregulation of a pathway in terms of the expression of its genes (see Methods for details), with respect to a reference sample. We take the lean group as reference sample for batch 1–4, and the normal tissue group for batch 9.

Figure 4a shows that all 16 pathways are deregulated in batches 1–4. More relevantly, it also shows that most pathways deregulated in obese patients are also deregulated in breast-cancer patients. Notice that in Fig. 4a samples are sorted by increasing PDS value, and hence clustering of the lean/obese or normal/tumor groups along a pathway indicates a strong deregulation. This result might support the claim that the pathway deregulation in breast cancer shares some elements with that of obesity. We would expect, however, the former to be broader and more intense, to the point of shadowing the later when combined. This is something that can be further investigated by inspecting samples of breast cancer from lean and obese subjects. Indeed, when computing PDS scores for batch 8 (404 samples of breast tumor with associated BMI data), we do not find significant changes in our score comparing obese with lean subjects. Unfortunately, we do not have associated normal tissue samples for batch 8, so the deregulation of tumor with respect to normal tissue cannot be verified in this case. As a further verification, we plot lean/obese and normal/tumor samples after a PCA transformation, see Supplementary Figure 5. In the case of Hs Inflammatory Response Pathway, compared to lean samples obese samples are closer to tumor samples, in agreement with the important role played by inflammation both in obesity and in cancer.

### Obesity signature and type 2 diabetes in adipocytes

**Fig. 5:**
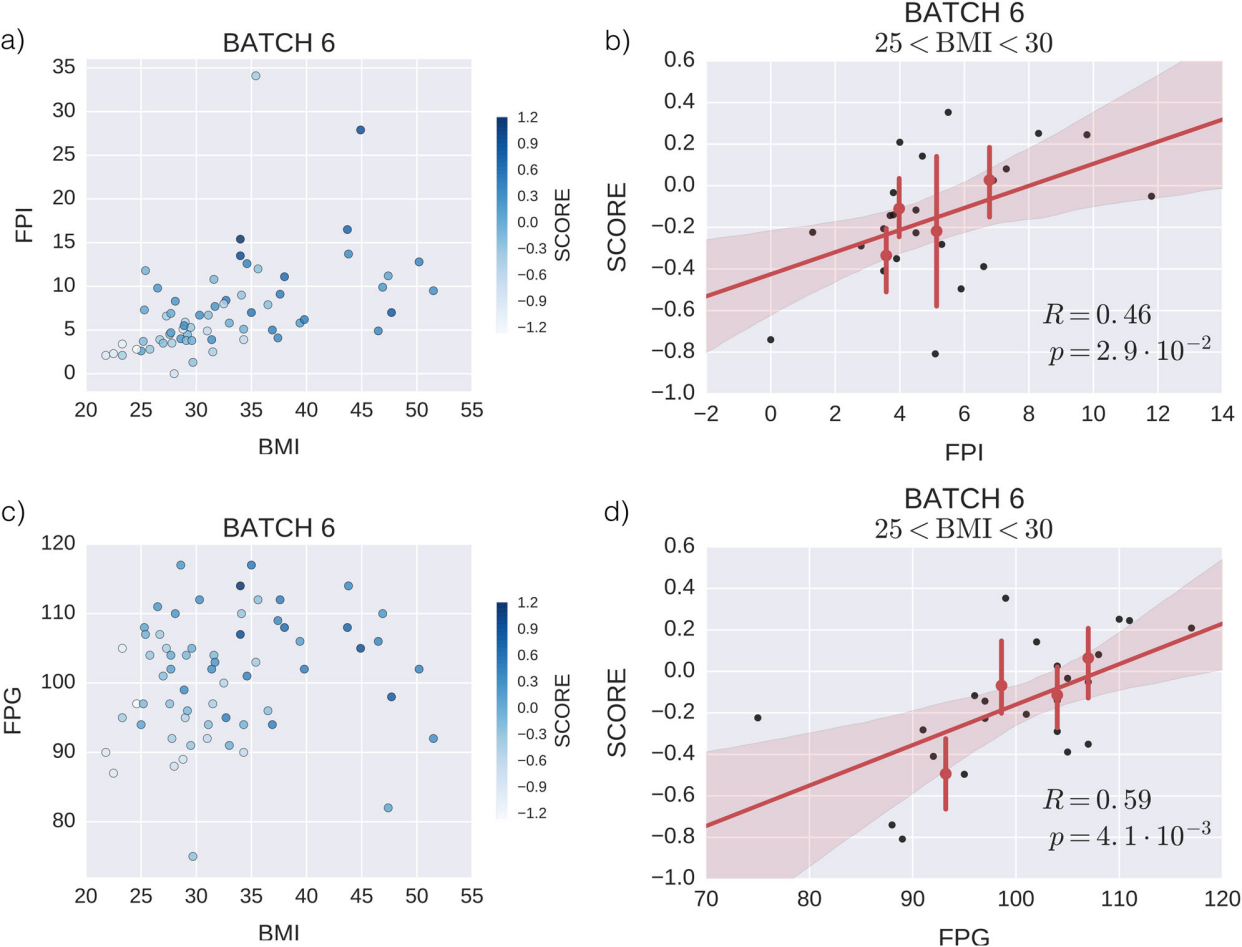
The obesity score correlates with fasting plasma glucose (FPG) and fasting plasma insulin (FPI) in overweight patients. **a**, **c** Scatter plot of FPI **a** and FPG **c** vs. BMI for all patients in Batch 6. Each point corresponds to a patient and is colored according to its obesity score. **b**, **d** Regression plot of obesity score vs. FPI in panel **b** and vs. FPG in panel **d**, showing only patients from Batch 6 categorized as overweight. *P*-values are computed via two-sided null hypothesis of 0 slope. Data is binned using percentiles and displayed with 95% CI *red error bars*, with a *red dot* marking the mean value of the in each bin. The *red solid line* is the fitted regression line, and the *red shaded area* corresponds to 95% CI of the regression line, computed via bootstrap.

More than 70% of obese subjects also suffer from diabetes.^2^ To assess whether our signature correlates not only with obesity but also with diabetes, we consider the data in batch 6 for which available clinical data includes BMI, fasting plasma insulin (FPI) and fasting plasma glucose (FPG). Figure 5b, d shows that our score correlates not only with BMI, but also with FPG (*R* = 0.59, *p* = 4.12 × 10^−3^) and FPI (*R* = 0.46, *p* = 2.88 × 10^−2^) when considering only overweight subjects with roughly the same BMI. For the case of FPG, Fig. 5c shows very clearly how the obesity score increases (marked with increasingly *darker blue points*) both when BMI increases (horizontal axis, left to right) and when FPG increases (vertical axes, bottom to top). Notice that BMI and FPG are not correlated in this case (*R* = 0.14, *p* = 0.29), and so we can conclude that our transcriptomic signature captures changes in two independent clinical traits.

### Transcriptomic signature of obesity and type 2 diabetes in monocytes and the effect of bariatric surgery

To asses if our signature is tissue-specific, and in particular to asses if a trace of our obesity signature could be detected in monocyte samples, we first analyze data from batch 10,^24^, see Supplementary Table 4 for details.^24^ This data set consists of paired subcutaneous adipose tissue (AC) and peripheral monocyte (MC) samples of 18 obese women, before and 3 months after bariatric surgery. As is clear in Supplementary Figure 3a, most of the genes in the signature display large changes in expression between AC and MC, with fold-change values as high as 10. In contrast, changes due to surgery lead to more moderate values, see also Supplementary Figure 6. This is in part to be expected, as gene expression is known to highly depend on tissue.

To further investigate these differences, we compare fold-change values of the obesity score genes with those of the rest of the genes. Supplementary Figure 6 shows that, indeed, the genes of the obesity score have fold-change values comparable to the rest of the genes (*p* = 0.49 for adipocytes, *p* = 0.17 for monocytes) when one looks at the effects of surgery (Supplementary Figure 6a, b), and that in contrast, they are significantly different (*p* = 1.61 × 10^−6^ before surgery, *p* = 8.21 × 10^−7^ after surgery) when one looks at differences between adipocyte and monocyte samples. In other words, most of the genes in our obesity score have tissue-specific expression patterns, and do not show important changes due to bariatric surgery. We have also computed a “monocytes obesity signature” using batch 12 for the sake of comparison. We find a set of 104 genes, of which two are in our original (AC) obesity score. Only one out of these two genes was already detected with our AC-MC co-expression data, see Supplementary Figure 3c. This suggests that the transcriptomes of monocytes and adipocytes are very well separated.

## Discussion

Recent literature shows an increase in the risk of some types of cancer, such as breast cancer, in obese subjects.^3^ It is, however, still unclear why obesity is associated to these diseases and if there is a gender-dependent effect in this association. It is also unclear if bariatric surgery, currently a very common procedure for severely obese subjects, might have any systemic effects, in particular in reducing the risk of developing cancer.

In the present paper, we approach these questions from the point of view of big data. One of the main problems to face is the fact that each biological study is intrinsically limited, be it due to the small numbers of patients involved or due to the heterogeneity of conditions and drug treatments among patients. Combining data from different studies should alleviate these problems but the presence of batch effects prevents straightforward merging of different data sets. Here, we solve the problem by combining SVD filtering with pathway deregulation analysis. In this way we reveal a robust transcriptomic signature of 38 genes that are differentially expressed in adipocytes coming from obese and lean subjects. Interestingly, the signature appears to be specific to the biological condition of obesity and is not linked to the gender of the subjects. The robustness of the signature has been confirmed on four independent data sets totaling almost 300 validation samples, as well as comparison with gene-level summary statistics from the Twins UK database.^22^ Additionally, when we compare subjects with similar BMI, between 25 and 30, we find that the score also correlates with the level of FPI or FPG, supporting the view that obesity is related to important complications such as type 2 diabetes.

A closer look at the genes of the signature reveals links to inflammation and immunity and well-known complications of obesity such as type 2 diabetes or fertility. From the three down-regulated genes (ST3GAL6, C12orf39, and CA3), ST3GAL6 is particularly interesting since a recent paper shows that the altered N-glycosylation of TNF-alpha treated adipocytes correlate with regulation of specific glycosyltransferases, such as the downregulation of ST3GAL6 sialyltransferase.^25^ Therefore, adipose inflammation associated with obesity modulates protein glycosylation, leading to an important biological deregulation.^25^ This kind of deregulation could lead to a broad effect on the biology of the cells. Carbonic anydrase III muscle specific (CA3), highly expressed in skeletal muscles, is also expressed by adipocytes.^26^ Interestingly, Lynch and colleagues show a decrease of adipose tissue CA3 in obese Zucker rats.^26^ Interestingly, these rats show peripheral insulin resistance, and adipose tissue hyper-responsiveness to the actions of insulin.^26^ To examine the possible role of insulin in obesity-dependent loss of adipose tissue CA3, Lynch and colleagues treat the obese Zucker rats with streptozotocin to induce diabetes, showing that the level of CA3 increases.^26^ Therefore from a therapeutic point of view, both ST3GAL6 and CA3 appear to be new and interesting possible targets for the treatment of obesity. If we look carefully at the role of the remaining 35 up-regulated genes in our signature, it is possible to identify some interesting genes that could be used as target for possible therapeutic interventions, while others are generically involved in many biological functions. In particular, CCDC80 and EMP3 are involved in the control of proliferation. The remaining 35 genes are all up-regulated. Among them we find Cathepsin S (CTSS) and GTPase of the immunity-associated protein 6 (GIMAP6), which are involved in inflammation and immunity: CTSS is a gene encoding for Catepsin S, a lysosomal cysteine proteinase that may participate in the degradation of antigenic proteins to peptides for presentation on major histocompatibility complex (MHC) class II molecules. Cathepsins S circulating levels have been found to correlate with BMI and triglycerides.^27^ Changes in weight due to dietary or bariatric surgery modulate either CTSS adipose tissue expression or Cathepsin S systemic circulating levels.^28^ GIMAPs (GTPases of the immunity-associated proteins), a family of small GTPases expressed prominently in the immune systems of mammals and other vertebrates, are known to play a role in modulating autophagy.^29^ We also find two biomarkers of the cerebral nervous system: CADM3 a synaptic cell adhesion molecule^30^ and SORBS2. An association between obesity and various neurological disorders has already been reported, including sleep apnea, anxiety, manic depressive disorders, increased risk of developing cerebrovascular accident, and other neurological disorders.^31^ Another interesting factor that is present in our signature is the transferrin receptor. It is known that iron homeostasis in obesity is impaired and in fact our signature highlights a key regulator of iron homeostasis.^32^ Finally, it is well known that fertility could be negatively affected by obesity.^33^ In our signature we find the FHL-5 gene which encodes a protein coordinately expressed with activator of cAMP-responsive element modulator (CREM) known to confer a powerful transcriptional activation function. In particular, CREM is known to act as a transcription factor essential for the differentiation of spermatids into mature spermatozoa. To conclude our analysis, there are also other genes which are more generic and are involved in many biological processes from inflammation, to cell proliferation to remodeling of the extracellular matrix.

By connecting genes to pathways, we find a set of 16 pathways (Supplementary Table 3) that can be grouped into three main categories: adhesion molecules which are involved in the interaction with the extracellular matrix and related intracellular signals (i.e., PI3K-AkT pathway); inflammation that can be involved in tumor development; and pathways connected to typical symptoms of obesity, from salivary secretion to digestive problems.

It is interesting to compare our signature with the results of integrative approaches.^7, 9^ In ref. 7, the authors identify causal genes for obesity in mice using a probabilistic Bayesian network approach that integrates DNA variation and expression data. They use liver and adipose co-expression data together with genetic loci related to obesity traits to identify a network of candidate genes, some of which were later experimentally confirmed as obesity causal genes using transgenic and knockout mice on fat diets.^9^ Focusing on the subset of their candidate genes that we could map to human genes present in our data, we find that COL1A2, EMP3, CTSS, BICC1, IFI27, SH3BGRL3, and COL6A1 are both in our obesity signature and in the their list of candidate genes (*p* = 4.10 × 10^−3^, hypergeometric test), supporting the consistency of our results with respect to previous integrative approaches.

We also explore the possibility to use transcriptomes obtained from peripheral monocytes, instead of adipocytes, and we find that tissue specific effects do not allow to reach any conclusions from monocyte sample. This highlights the well-known fact that transcriptomic signatures are generally tissue-specific, and special care must be taken when used against diverging tissues.

In conclusion, we show that a combined analysis of gene expression data present in the literature allows to draw a clear picture of the deregulation associated with obesity and the relations with type 2 diabetes and cancer. Interesting markers come out from our analysis and they can easily be used for prognostic purposes and followed during specific drug or dietetic regiment. The strength of our work comes from the use of appropriate filtering and noise reduction methods that allow to mitigate batch effects. This general strategy can be naturally extended to other pathological conditions, providing a clear avenue to analyze the massive amount of data accumulating in the biomedical literature. Improvement on our results could be obtained using larger cohorts and more precise measurements of the fat mass, such as those obtained by Dual-energy X-ray absorptiometry (DEXA) or echoMRI.

## Methods

### Batch effects removal

We use the SVD technique introduced in ref. 19. SVD correction consists in transforming the space of *N*-genes × *M*-arrays to a new space of *L*-eigengenes × *L*-eigenarrays with *L* = min{*M*, *N*}. In practice, one has *L* = *M* because the number of genes almost always exceed the number of samples available.

The SVD decomposition of a matrix *X* can be written as follows:

1 $$X = U \Sigma {V^T} $$

where Σ is a diagonal matrix with entries *λ*_1_ > *λ*_1_ > … *λ*_*L*_ > 0. The column vectors *u*_1_,…,*u*_*L*_ of *U* are linear combinations of genes, called eigengenes, while the row vectors *v*_1_,…,*v*_*L*_ of *V* ^*T*^ are linear combinations of arrays, thus called eigenarrays. Eigengenes might be associated with a biological process, while eigenarrays would correspond to cellular phenotypes. The idea behind SVD-correction is to filter out those eigengenes that are inferred to correspond to batch effects rather than to a true biological process. See ref. 19 for details.

We adapt and apply this technique for the more involved situation we are interested in, that of merging *K* batches with expression matrices *X* ^(1)^,…,*X* ^(*k*)^. In particular, we have developed our own two-step SVD batch-effect removal method, as follows:

1. 1st SVD-filtering Step: For each data set *k* = 1,…,*K*, filter out the first $\ell - 1$ eigengenes $u_1^{\left( k \right)}, \ldots ,u_{\ell - 1}^{\left( k \right)}$ that do not contain useful information for our phenotype of interest, obtaining a modified expression matrix ${\hat X^{\left( k \right)}}$,
  2 $${\hat X^{\left( k \right)}} = {X^{\left( k \right)}} - \mathop {\sum}\limits_{i = 1}^{\ell - 1} {\lambda _i^{\left( k \right)}\left( {u_i^{\left( k \right)} \cdot v_i^{\left( k \right)}} \right)} $$
  Notice that the value of $\ell$ is *k*-dependent, $\ell \equiv {\ell ^{\left( k \right)}}$.
2. Merging Step: Merge the *K* matrices ${\hat X^{\left( k \right)}}$. Notice that after step 1, the columns of ${\hat X^{\left( k \right)}}$ still represent genes. Thus the matrices can be aligned without further complications, creating a new matrix *Y*,
  3 $$Y = {\left[ {{{\hat X}^{\left( 1 \right)}}, \ldots ,{{\hat X}^{\left( K \right)}}} \right]^T}$$
3. 2nd SVD-filtering Step: Filter out the first $\ell - 1$ eigengenes ${u_1}, \ldots ,{u_{\ell - 1}}$ that do not contain useful information for our phenotype of interest in the newly created matrix *Y*, obtaining thus a new expression matrix $\hat Y$,

4 $$\hat Y = Y - \mathop {\sum}\limits_{i = 1}^{\ell - 1} {{\lambda _i}\left( {{u_i} \cdot {v_i}} \right)} $$

In both SVD-filtering steps, the value(s) of $\ell$ is set to

5 $$\ell \equiv {\rm{argma}}{{\rm{x}}_i}\left\{ { - \log \left( {p_i^{{\rm{KS}}}} \right)} \right\} = {\rm{argmi}}{{\rm{n}}_i}\left\{ {p_i^{{\rm{KS}}}} \right\}$$

where $p_i^{{\rm{KS}}}$ is the *p*-value of a 2-sample Kolmogorov–Smirnov test comparing the expression of the *i*-th eigengene between lean and obese samples. In practice, the expression values of the *i*-th eigengene among samples corresponds to the *i*-th row of the matrix *U* ^*T*^ *X*.

The whole process can be summarized as follows:

6 $$ \left\{ {{X^{\left( k \right)}}} \right\}_{k = 1}^K\mathop{\longrightarrow}\limits^{{{\rm{SVD}} - {\rm{filtering}}}} \left\{ {{{\hat X}^{\left( k \right)}}} \right\}_{k = 1}^K\mathop{\longrightarrow}\limits^{{{\rm{}}  {\rm{merging}}}} \,\,Y\mathop{\longrightarrow}\limits^{{{\rm{SVD}} - {\rm{filtering}}}}\hat Y,$$

In plain words, the first filtering step makes sure that the phenotype of interest (obesity in our case) is “the strongest effect” on each of the batches. After merging, batch effects appear, and the second filtering step removes all eigengenes whose strength (measured by the corresponding eigenvalue *λ*) is larger than the eigengene that better discriminates the phenotype of interest (measured by a KS test).

### Obesity score

We define the obesity score *S* as

7 $$S \equiv \mathop {\sum}\limits_{k = 1}^n {{\alpha _{i\left( k \right)}}{X_{ji}}} $$

where *α*_*i*(*k*)_ is the coefficient of *k*-th largest absolute value of the first principal component of batches 1 to 4, after batch-effects have been removed, *X*_*ji*_ is the log2 expression level of gene *i* in sample *j* in any batch. We fix a value of *n* = 38 by imposing that all genes included in the score are beyond a 5*σ* range with respect to the coefficients of a random vector. This corresponds to a *p*-value of 5.70 × 10^−7^ (FDR equivalent: 1.90 × 10^−3^). See Table 1 for the list of the 38 genes and their associated coefficients, and Supplementary Figure 1 for a summary of their main characteristics among the different batches. Obesity scores are displayed as mean-centered values in all figures.

In summary, the obesity score is defined as the a linear combination of the log2 expression of 38 genes. These 38 genes and their coefficients are computed only once, using batches 1 to 4 after removing batch effects, and kept fixed for the rest of the analysis.

### Calculation of pathways over-represented in score genes

To determine pathways significantly over-represented in the set of 38 genes in the obesity score, we use a hypergeometric null model. In particular, given a pathway with *K* genes, we compute its associated *p*-value as the probability of finding *k* or more of its genes in a random choice of *n* = 38 genes among a total of *N* = 13684 available genes. To compute the value of *K*, we only take into account the 13684 genes that result from merging batches 1 to 4, as these are the ones available when the score is defined. We restrict to pathways with *k* ≥ 2, finding a total of 16 pathways. Next, we assign a family-level *p*-value to this set of 16 pathways by empirically computing the distribution of the number of pathways with at least two genes in common with the obesity score. The *p*-value of 0.012 reported in the main text and in Supplementary Table 3 corresponds then to the probability of finding 16 or more pathways with at least two genes in common with the score, under the null hypothesis and indicates that, as a whole, the set of 16 pathways is statistically significant.

### Calculation of PDS

PDS were first introduced by Drier et al., 2013^20^ as a way of quantifying the overall deregulation of a given pathway, with respect to a reference sample. They are computed by fitting a non-parametric, non-linear one-dimensional curve through the “middle” of the transcriptomic data, in the subspace generated by the genes of that pathway. In practice, this is usually done via the *principal curve* algorithm,^34^ although other procedures would be acceptable. We follow the steps carefully explained in ref. 20, except for the following modification: the value of 0 is placed at the mean value of the reference sample, instead of at the extremal point of the curve. This modification can alter the values as computed in ref. 20 only by a linear shift, and makes the results more robust to the variability of the reference sample.

### Code availability

A repository with all code used to generate the results of this paper is available at https://github.com/ComplexityBiosystems/obesity-score. A sandalone python package implementing the SVD batch-effects removal method is also available, see https://github.com/ComplexityBiosystems/SVDmerge

### Data availability

All relevant data are available at the Gene Expression Omnibus (GEO) under accession numbers GSE2508, GSE26637, GSE27949, GSE48964, GSE62117, GSE64567, GSE33526, GSE78958, GSE65540, GSE66306, and GSE32575 (see Supplementary Table 4 for details). In addition, data for batch 11 was obtained from the Bgee Gene Expression Database^35^ and can be publicly accessed at http://bgee.org/. Summary statistics from the Twins UK dataset used in Supplementary Figure 2 can be accessed at http://expression.kcl.ac.uk/phenoexpress/1/.

## Electronic supplementary material

Supplementary materials
