# Supplementary material for: Integrative analysis of pathway deregulation in obesity
Source: NPJ Syst Biol Appl. 2017 Jun 30;3:18. doi: 10.1038/s41540-017-0018-z (PMC5493646; doi:10.1038/s41540-017-0018-z)
Supplement: Supplementary file 1 — Supplementary materials [file 41540_2017_18_MOESM1_ESM.pdf]

Supplementary Information: Integrative analysis of pathway deregulation in obesity

Supplementary Methods

Computation of empirical p-values for number of overlapping genes in male/female-only signatures

We use gender labels in batches 1-4 to compute male-only and female-only signatures. To compute the p-value associated to the number of overlapping genes, we proceed as follows:

For $i = 1, \dots, N$:

1. Create two random non-intersecting groups A, B with number of elements matching those of the real male/female groups in batches 1–4.
2. Compute an A-only signature and a B-only signature in exactly the same way as done with the male/female groups.
3. Compute the number of overlapping genes k_i between the A and B signatures.

This way we obtain a sample of $\{k_1, \dots, k_N\}$ of the number of overlapping genes, generated under the null hypothesis of independence of gender and respecting the sizes of the groups to account for finite-size effects. Notice that in this situation, a signature that depends on the A, B groups leads to a smaller overlap and hence to small values of our statistic k. Therefore, the p-value associated to finding k overlapping genes in the male/female signatures is

$$p(k) = \frac{|\{k_i : k_i \leq k\}|}{N}$$

In our case, we obtained $k = 18$ overlapping genes, which corresponds to a p-value of 0.874. The null hypothesis would have been rejected (with significance level of 0.05) if we had found 4 or less overlapping genes.

Transcriptomic data sets

We gather publicly available transcriptome data sets form the Gene Expression Omnibus (GEO), The Cancer Genome Atlas (TCGA) and the Bgee databases. For batches 1 to 8, subjects are categorized as either *lean* (BMI$<$ 25), *overweight* (25 $\leq$BMI$<$ 30) or *obese* (30 $\leq$BMI) if the exact body mass index (BMI) is known. Otherwise, the available categorical metadata is used (e.g., samples tagged simply as “lean” or “obese”).
We apply the following pre-processing steps to all transcriptomic data: 1) Probes containing missing values are excluded from the analysis. 2) Probes are mapped to Entrez ID labels if they are available in the associated platform. Otherwise the David portal is used to convert the available labels to Entrez ID labels. 3) Values corresponding to raw expression counts or gene expression intensity are log2 transformed (if necessary). 4) Probes mapping to the same Entrez ID label are averaged out. 5) Probes that cannot be mapped to a unique Entrez ID label are excluded from the analysis, as well as those that cannot be mapped to any Entrez ID label at all. 6) We apply a simple $\mathscr{L}_1$ normalization in linear space, imposing that the sum of expression of all genes is constant among samples. After these steps, each data set or batch is represented by a single expression matrix X. Each entry X_{ij} represents the $\log 2$ of the expression intensity of gene i in sample j.

Batch 1

Abdominal subcutaneous adipocytes from 20 lean (BMI 25±3 $\mathrm{kg/m^2}$) and 19 obese (BMI 55±8 $\mathrm{kg/m^2}$) non-diabetic Pima Indian subjects.

Batch 2

Subcutaneous adipose tissue from 5 lean insulin-sensitive (BMI 22±0.7 $\mathrm{kg/m^2}$) and 5 obese insulin-resistant (BMI 32.5±1.7 $\mathrm{kg/m^2}$) non-diabetic Caucasian women. Two samples per subject, one at fasting and one after 3h hyperinsulinemia, totaling 20 samples (10 lean, 10 obese) as reported in Supplementary Table 4.

Batch 3

Subcutaneous adipose tissue from 6 lean (BMI 22.13±2.7 $\mathrm{kg/m^2}$) and 17 obese (BMI 37.28±4.7 $\mathrm{kg/m^2}$) subjects, categorized according to their glucose tolerance status, see Supplementary Table 1. Ten overweight subjects were discarded from the analysis to match batches 1, 2 and 4, where this category is not present. The original study focuses on miRNAs, but provides *“a novel transcriptomics database of EXIQON and Affymetrix adipocyte profiles to facilitate data mining”*.[12] We are grateful to Keller *et al* for providing the Affymetrix profiles.

Batch 4

Adipose stem cells from subcutaneous abdominal white adipose tissue from 3 lean (BMI $22.26 \pm 0.88 \mathrm{kg/m^2}$) and 3 obese (BMI $44.44 \pm 1.29 \mathrm{kg/m^2}$) subjects. Obese subjects showed significantly elevated glucose, creatinine levels and total cholesterol/high density lipoprotein cholesterol (HDL) ratio; as well as lower HDL cholesterol levels, see[15] for details.

Batch 5
Subcutaneous adipose tissue samples from 42 lean (BMI $22.28 \pm 2.13\mathrm{kg/m^2}$), 39 overweight (BMI $27.43 \pm 1.56\mathrm{kg/m^2}$) and 21 obese (BMI $33.28 \pm 2.78\mathrm{kg/m^2}$) samples, corresponding to 21, 28 and 12 unique subjects respectively, see Supplementary Table 2. Subjects are either HIV-positive in one of three antiretroviral therapy regimes (zidovdine and lamivudine (AZT); tenofovir and emtricitabine (TDF) or abacavir and lamivudine (ABC) as well as efavirenz for all three treatments), or HIV-negative healthy controls. Samples where taken before the start of the treatment, 6 months in treatment and 18-24 months in treatment. BMI was measured at each time point, and BMI relative variations for a fixed subject as large as $\sim 30\%$ are observed, with $\sim 5-10\%$ being typical. As one expects variations in BMI to be reflected in the transcriptome, all samples are included in the analysis and considered independent of each other. We have checked that the 3 treatments and the control group are well balanced across the different BMI status groups, see Supplementary Table 2.

Batch 6
Adipose tissue samples from 5 lean (BMI $23.10 \pm 1.05\mathrm{kg/m^2}$), 24 overweight (BMI $27.65 \pm 1.50\mathrm{kg/m^2}$) and 35 obese (BMI $37.61 \pm 6.17\mathrm{kg/m^2}$) Mexican American subjects from the Veterans Administration Genetic Epidemiology Study (VAGES). Subjects in the VAGES were ascertained on at least 2 siblings and one parent affected with type-II diabetes (T2D). The analyzed samples have several associated clinical traits, including body mass index, fasting plasma glucose and fasting plasma insulin.

Batch 7
Reduction mammoplasty tissue from 14 lean (BMI 23.63 ± 0.87 $\mathrm{kg/m^2}$), 28 overweight (BMI 27.42 ± 1.41 $\mathrm{kg/m^2}$) and 30 obese (BMI 36.53 ± 4.85 $\mathrm{kg/m^2}$) subjects.

Batch 8
Primary breast tumor samples from 131 lean, 131 overweight and 142 obese subjects from the Clinical Breast Care Project. Unfortunately, after categorizing subjects in the three BMI status categories, the original continuous BMI variable was not reported in.[17] A total of 20 samples with unknown BMI were discarded from the analysis. Samples are also classified by tumor grade, stage and sub-type.

Batch 9
Breast invasive carcinoma (BRCA) cohort of The Cancer Genome Atlas (TCGA) project, accessed on 21/07/2015. For subjects with both normal tissue and tumor principal samples, only the first was kept. A total of 114 normal tissue and 979 tumor principal samples were analyzed.

Batch 10
Adipose and peripheral monocytes paired samples of 18 obese women, before and 3 months after bariatric surgery.

Batch 11
Samples pulled from the Bgee[26] database, for species Homo Sapiens and tissues "adipose tissue" (N=275) and "blood" (N=488). These samples are used to confirm gene-coexpression patterns inferred in batch 10, see Supplementary Figure 3.

Batch 12
12 samples from 6 lean subjects ((BMI $20.3 \pm 0.5\mathrm{kg/m^2}$)) and 36 samples from 18 obese subjects, before (BMI $45.1 \pm 1.4\mathrm{kg/m^2}$) and 3 months after bariatric surgery (BMI $37.5 \pm 1.3\mathrm{kg/m^2}$).

Pathways data sets
We download and parse data form four pathways databases: KEGG, Biocarta, Wikipathways and pantherDB. In addition, we also consider "pathways" constructed via miRNA co-targeting, using the miRTarBase database. Biocarta pathways were downloaded and parsed from the Cancer Genome Anatomy Project (CGAP). The David portal was used to match Unigene to Entrez ID gene labels. KEGG pathways were obtained via the KEGG submodule of the bioservices python module. Wikipathways pathways were downloaded from the project site on 30/11/2015. In this case, we restrict the analysis to those given in terms of Entrez ID labels, covering 83% of the database. miRNA pathways are constructed using the microRNA-target interaction database miRTarBase. In particular, each microRNA gives rise to a "pathway", formed by the set of genes that it targets (only experimentally-validated interactions are included). The pathways at pantherDB.org are given in terms of proteins, complexes and other small molecules instead of genes. We use the Uniprot database to match genes to proteins, keeping only those pathways consisting of three or more genes for which at least 70% of the proteins where matched to at least one Entrez ID gene.

Software
All data processing, statistical analysis and figures rendering are handled with the following python open-source libraries: numpy, scipy, pandas, GEOparse, scikit-learn, seaborn and bioservices.

Gene names mining for Supplementary Figure 1

To automatically detect gene mentions in the original references of all 8 batches, we expand our list of 38 genes by also including previous official gene symbols and known synonyms pulled from the HUGO Gene Nomenclature Committee (HUGO) database on June 13, 2016. We then match this expanded list with a text-only version of the references, which we create using the pdftotext utility from the poppler library. We also check that our script is able to find genes that are in the references but not in our obesity score.

Supplementary Figures

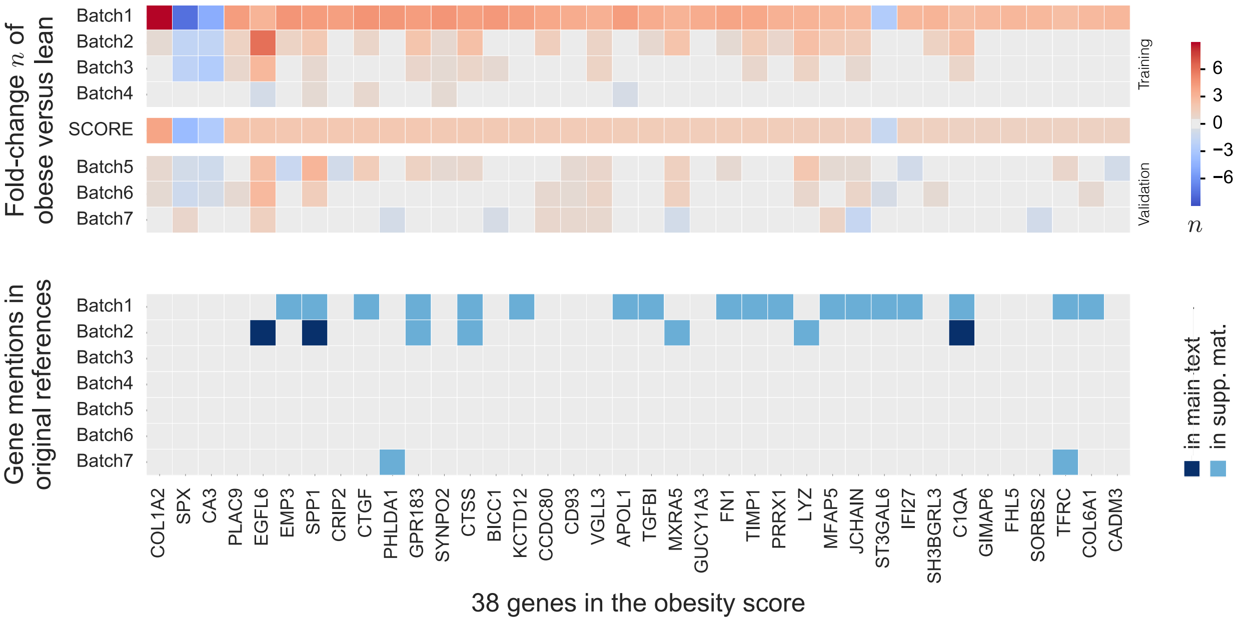

Supplementary Figure 1. **The 38 genes in the obesity score.** (Top panel): Fold-change value n between lean and obese patients, in log2 units. Red (blue) colored squares correspond to genes overexpressed (underexpressed) in obese with respect to lean. Darker coloring means larger fold-change absolute value. A row representing the coefficients (in arbitrary scale) of the genes in the score was added for comparison. All values between -0.5 and 0.5 are colored gray to enhance visualization of the panel. (Bottom panel): Gene mentions in the original references associated to each batch, see Table 1 in the main text for details. We distinguish mentions in the main text (dark blue) from mentions in the supplementary material (lighter blue), which can include long multi-page tables with hundreds of genes. Batches 1 to 4 were used to construct the obesity score, while batches 5,6 and 7 are independent validation data sets.

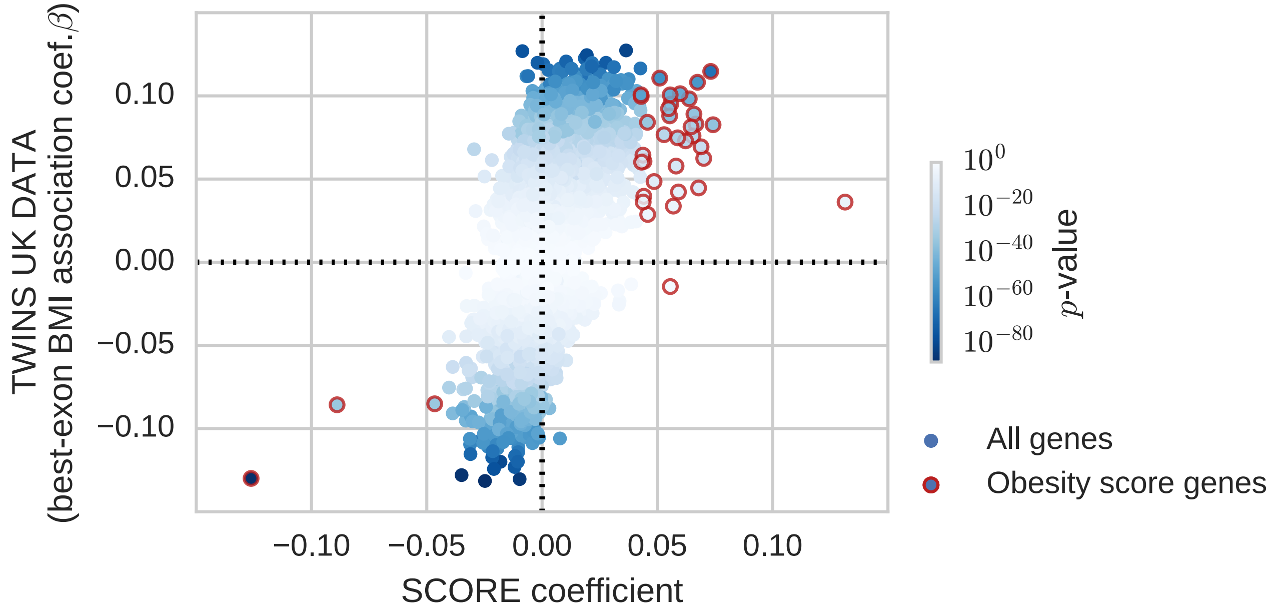

Supplementary Figure 2. Comparison of the obesity score coefficients with Twins UK summary statistics. The x-axis shows the coefficient of each gene in the first principal component of batches 1–4 after SVD merging. The y-axis show the best-exon association coefficient with BMI in the Twins UK dataset.[22] Coloring corresponds to p-values as reported in.[22] Notice that this comparison is only provided as a sanity test, and no perfect agreement is expected at all as the quantities being compared are fundamentally different: the values in the y axis come from a exon-by-exon fit measuring the association between BMI and expression, see for details, while our values are obtained simply by computing the first principal component of batches 1–4 after SVD merging.

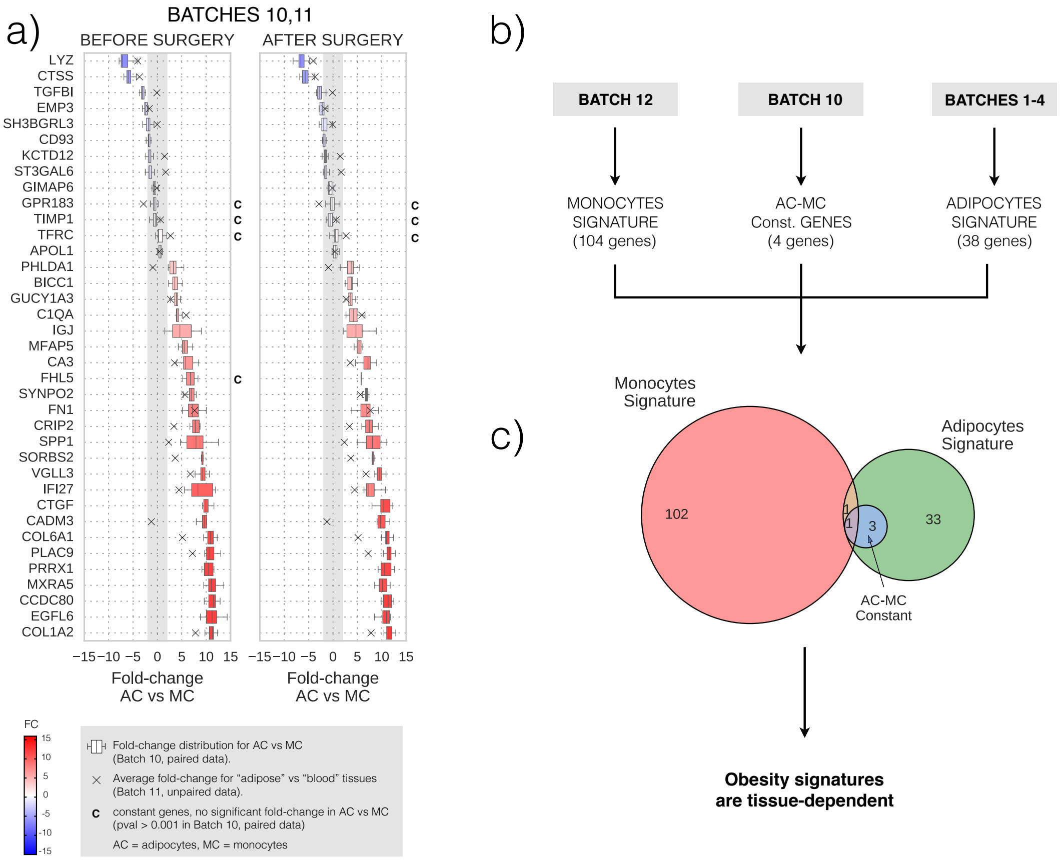

Supplementary Figure 3. **Tissue-specific expression of the genes of the signature**. (a) Fold-change expression of subcutaneous adipose tissue samples (AC) with respect to paired peripheral monocytes samples (MC) of 18 obese women, before and 3 months after bariatric surgery (Batch 10). Blue-to-red colored boxplots show the distribution of fold-change values, for each gene of the signature, among the 18 subjects. The **C** sign mark genes for which the null hypothesis of same average expression on AC and MC cannot be rejected, at a significance level of 0.001 and using a t-test for paired data. The cross signs correspond to the average fold-change of “adipose” versus “blood” tissues in the Bgee[26] database (Batch 11, unpaired data), and are provided only as comparison. The vertical gray strips mark a region of fold-change values between -2 and 2. (b) Panel describing how the original, adipose-tissue derived signature is modified to then be used with monocytes data sets. (c) Boxplots of the MC-adapted score against BMI categories (Batch 12). The adapted score is able to detect a trace of obesity in peripheral monocytes data, if constrained to **C** genes, i.e. to genes with similar expression patters in AC and MC tissues. The reported p-value of 5.5×10^{-5} is computed with a Kolmogorov-Smirnov test of the lean versus obese before surgery populations. (d) Boxplots of the MC-adapted score against type II diabetes categories (Batch 13). The adapted score is able to distinguish obese diabetic from obese non-diabetic subjects.

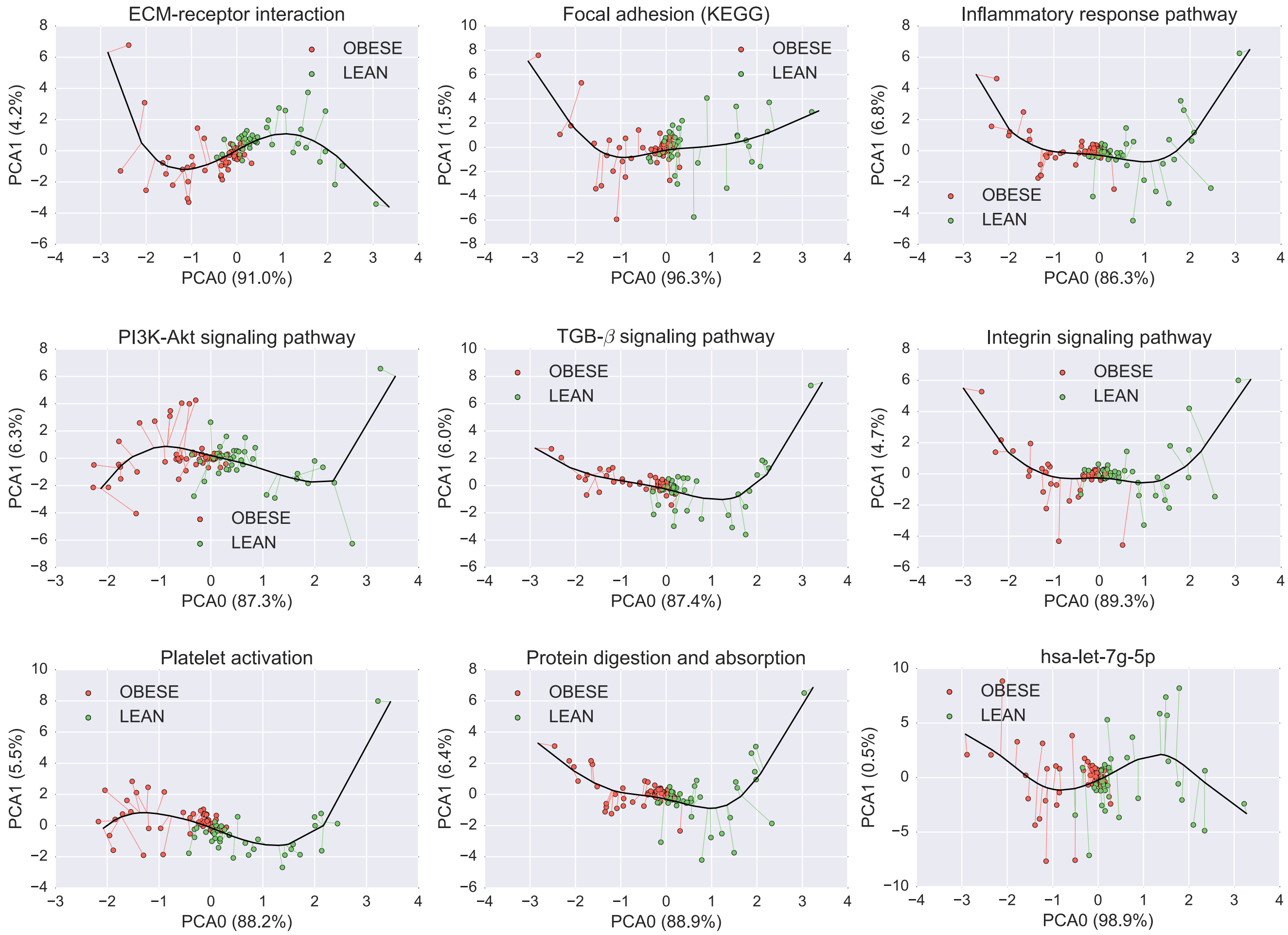

Supplementary Figure 4. Deregulation of pathways in obese patients.. Projection of batch 1-4 samples, shown with red (obese) and green (lean) dots, onto the principal curve (black line) that is used to define Pathway Deregulation scores. We show nine pathways from Table 3 in the main text. Auxiliary thinner lines connecting the unprojected with the projected data are plotted to ease visualization. For the purposes of this visualization only, all data is further projected onto its first two principal components, PCA0 and PCA1. Axis labels display the ratio of explained variance.

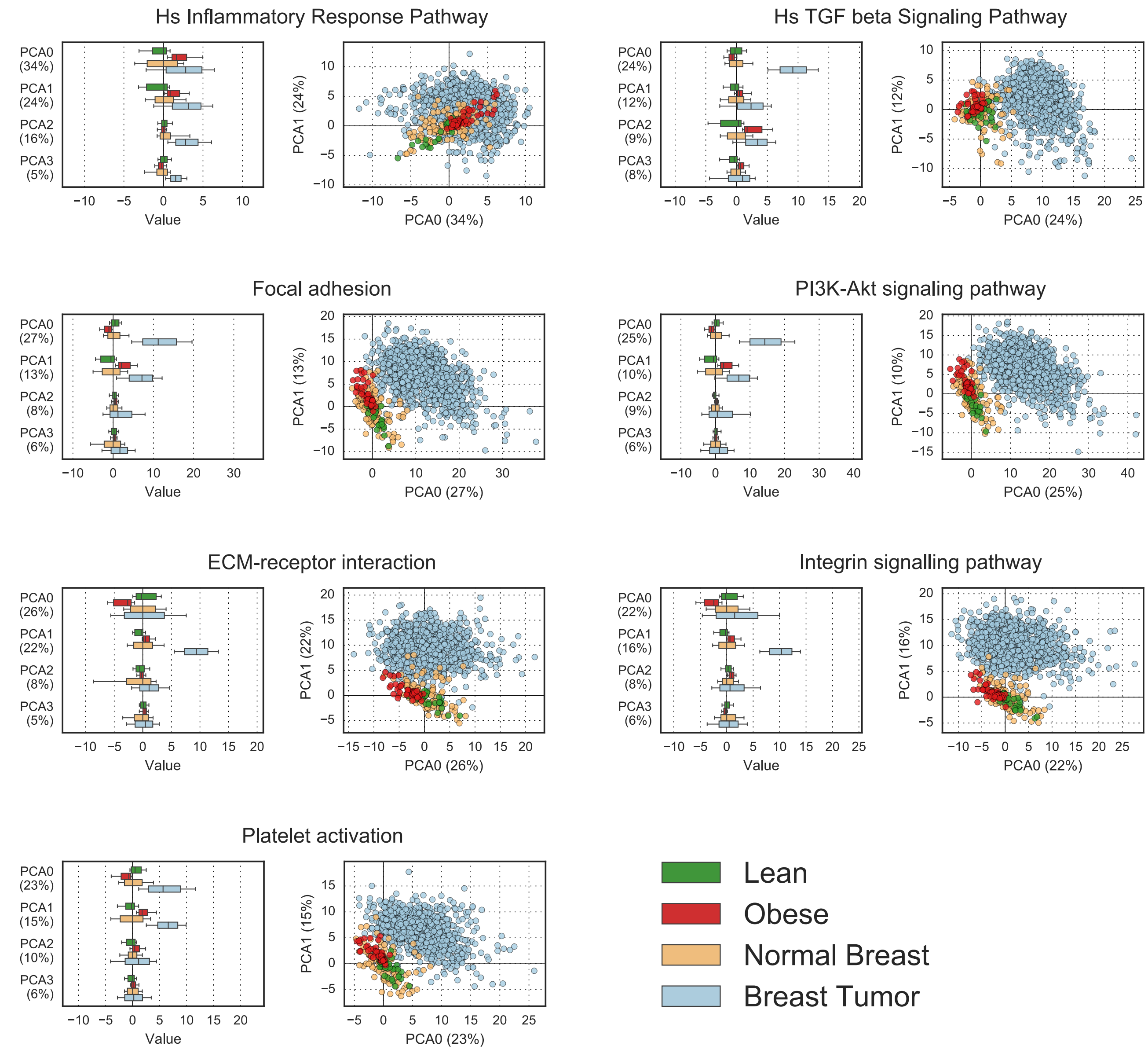

Supplementary Figure 5. Pathway-based PCA projection of obesity and cancer samples. Scatter plots and boxplots displaying the first few components of a PCA projection of seven pathways for both lean/obese samples from batch 1–4 and normal/tumor samples from TCGA. The panel shows that, compared to lean samples, obese samples tend to be closer to tumor samples, indicating similar deregulation patterns between obesity and cancer. Data was expressed as fold-change of obese with respect to lean or tumor with respect to normal before the PCA transformation. Projected data was median-centered on the "Normal Breast" group to enhance visualization of the panel.

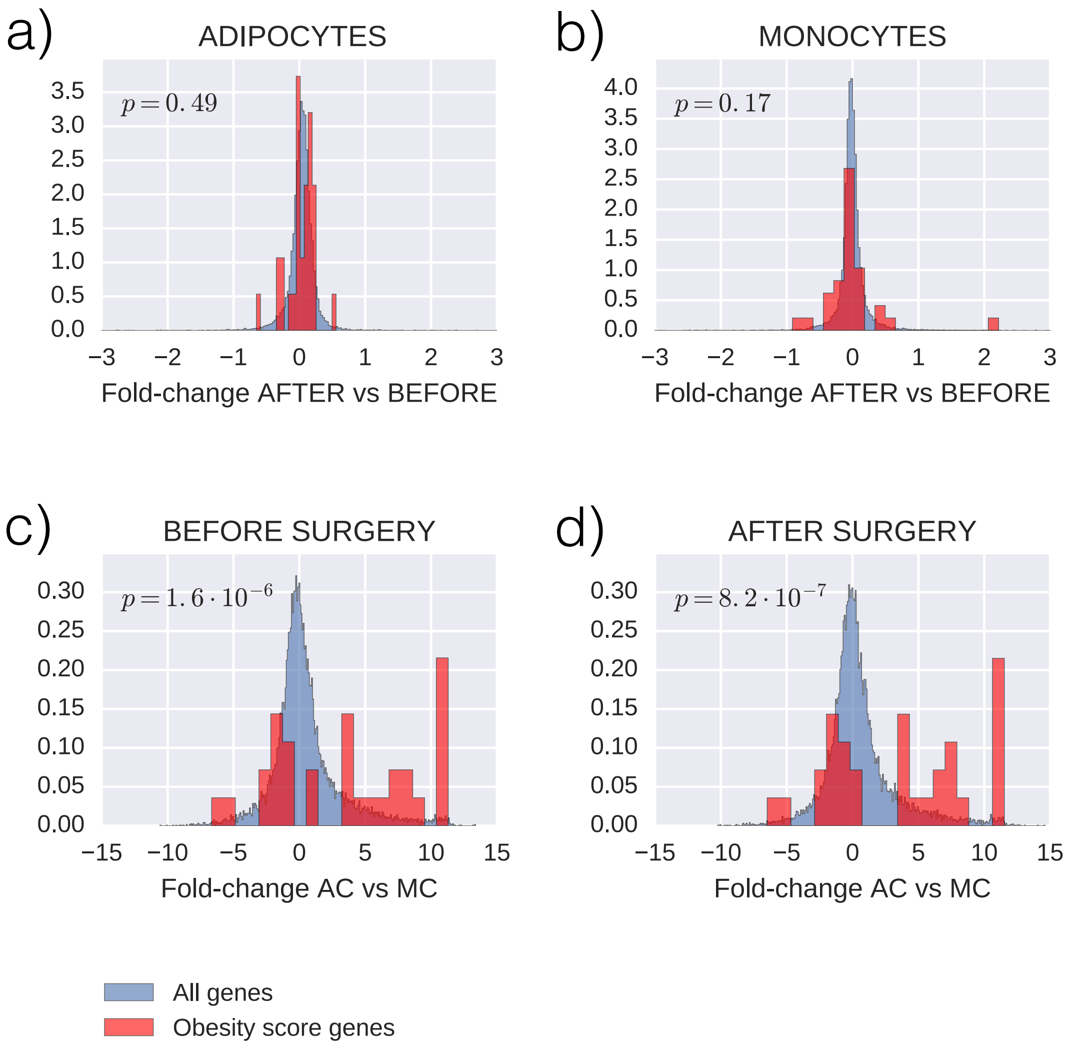

Supplementary Figure 6. Effects of bariatric surgery on adipocytes and monocytes gene expression. Top row: Distribution of fold-changes after bariatric surgery with respect to before bariatric surgery, for adipocytes (a) and monocytes (b). The blue shaded area shows the distribution among all genes, while the red one includes only the 38 genes in the obesity score. Bottom row: Same, computing fold-change values of adipocytes samples with respect to monocytes samples, both before (c) and 3 months after bariatric surgery. All p-values in the panel are computed with a Kolmogorov-Smirnov test comparing the two plotted distributions.

Supplementary tables

	NGT	IGT	DM	**Total**
Lean	3	2	1	6
Obese	6	6	5	17
Total	9	8	6	23

Supplementary Table 1. Number of samples per BMI status and glucose tolerance status in batch 3. NGT = Normal Glucose Tolerance, IGT = Impaired Glucose Tolerance, DM = Diabetes Mellitus

	ABC	AZT	TDF	NEG	**Total**
Lean	4(3)	13(5)	19(7)	6(6)	42(21)
Overweight	9(7)	11(8)	13(7)	6(6)	39(28)
Obese	6(5)	7(2)	5(2)	3(3)	21(12)
Total	19(15)	31(15)	37(16)	15(15)	102(61)

Supplementary Table 2. Number of samples (parenthesis, subjects), for each of the four treatments and each BMI status. See description of batch 5 for details on treatments.

Pathway	Database	p-value	k	K	Genes in obesity score
miRNA targets in ECM & membr. recpt.	Wiki	1.90×10^{-7}	3	19	COL6A1, COL1A2, FN1
ECM-receptor interaction	KEGG	1.88×10^{-6}	4	75	COL6A1, SPP1, COL1A2, FN1
hsa-let-7g-5p	miRNA	8.79×10^{-6}	2	15	COL1A2, FN1
Inflammatory response pathway	Wiki	7.62×10^{-5}	2	30	COL1A2, FN1
Focal adhesion	KEGG	1.35×10^{-4}	4	181	COL6A1, SPP1, COL1A2, FN1
Integrin signal ling pathway	pantherDB	5.30×10^{-4}	3	136	COL6A1, COL1A2, FN1
hsa-miR-124-3p	miRNA	5.49×10^{-4}	2	58	PRRX1, CTGF
PI3K-Akt signaling pathway	KEGG	8.21×10^{-4}	4	268	COL6A1, SPP1, COL1A2, FN1
Salivary secretion	KEGG	9.12×10^{-4}	2	69	GUCY1A3, LYZ
Protein digestion and absorption	KEGG	1.07×10^{-3}	2	73	COL6A1, COL1A2
Focal Adhesion	Wiki	1.17×10^{-3}	3	168	SPP1, COL1A2, FN1
HIF-1 signaling pathway	KEGG	1.35×10^{-3}	2	79	TFRC, TIMP1
Amoebiasis	KEGG	2.43×10^{-3}	2	97	COL1A2, FN1
Phagosome	KEGG	2.97×10^{-3}	2	104	CTSS, TFRC
TGF-β signaling pathway	Wiki	3.30×10^{-3}	2	108	COL1A2, FN1
Platelet activation	KEGG	3.47×10^{-3}	2	110	GUCY1A3, COL1A2

Supplementary Table 3. Pathways with at least 2 genes in common with the obesity score. P-values are computed using the hypergeometric test. K is the number of genes in the pathway, while k is the number of genes both in the pathway and the obesity score. The family-level p-value for the set of sixteen pathways is 0.012, see SI for details.

Batch	Lean	Overweight	Obese	Total	Gender	BMI	Tissue	Accession code	Ref.
Batch 1	20	0	19	39	19 M / 20 F	categorical	Adipose	GSE2508	10
Batch 2	10	0	10	20	All F	categorical	Adipose	GSE26637	11
Batch 3	6	(10)	17	23	Unknown	numerical	Adipose	GSE27949	12
Batch 4	3	0	3	6	Unknown	categorical	Adipose	GSE48964	13
Batch 5	42	39	21	102	49 M / 53 F	numerical	Adipose	GSE62117	14
Batch 6	5	24	35	64	19 M / 45 F	numerical	Adipose	GSE64567	15
Batch 7	14	28	30	72	All F	numerical	Normal breast	GSE33526	16
Batch 8	131	131	142	404	All F	categorical	Breast tumor	GSE78958	17
Batch 9	–	–	–	979	All F	none	Breast tumor	–	23
	–	–	–	114	All F	none	Normal breast	–	23
Batch 10	0	0	18	18	All F	categorical	Adipose	GSE65540	24
	0	0	18	18	All F	categorical	Monocytes	GSE66306	24
Batch 11	–	–	–	275	Unknown	none	Adipose	–	26
	–	–	–	488	Unknown	none	Blood	–	26
Batch 12	12	0	36	48	All F	categorical	Monocytes	GSE32575	18

Supplementary Table 4. Basic information of all 12 batches: number of samples per BMI status and gender, type of BMI information available (either categorical, numerical or none), GEO/ArrayExpress accession code (if available) and associated reference.
